# Anti-hypertensive effect of a novel angiotensin II receptor neprilysin inhibitor (ARNi) -S086 in DSS rat model

**DOI:** 10.3389/fcvm.2024.1348897

**Published:** 2024-02-14

**Authors:** Jingchao Sun, Ying Xiao, Wenjie Xu, Wei Xing, Frank Du, Maozhi Tian, Danqi Xu, Yihua Ren, Xin Fang

**Affiliations:** ^1^ R&D Center, Shenzhen Salubris Pharmaceutical Co., Ltd., Shenzhen, Guangdong, China; ^2^ iBHE, Tsinghua Shenzhen International Graduate School, Shenzhen, Guangdong, China; ^3^ Pharmacology Department, WuXi AppTec (Shanghai) Co., Ltd., Shanghai, China

**Keywords:** angiotensin receptor-NEP inhibitor (ARNi), hypertension, neprilysin inhibitor, angiotensin receptor blocker (ARB), natriuresis, diuresis

## Abstract

**Introduction:**

Angiotensin receptor-neprilysin inhibitor (ARNi), comprised of an angiotensin receptor blocker (ARB) and a neprilysin inhibitor (NEPi), has established itself as a safe and effective intervention for hypertension. S086 is a novel ARNi cocrystal developed by Salubris for the treatment of heart failure and hypertension.

**Methods:**

Dahl Salt Sensitive (DSS) hypertensive rat model and telemetry system were employed in this study to investigate the anti-hypertensive efficacy of S086 and compare it with the first ARNi-LCZ696.

**Results and discussion:**

The study showed that oral administration of S086 dose-dependently lowered blood pressure (*P* < 0.001). The middle dosage of S086 (23 mg/kg) exhibited efficacy comparable to LCZ696 (68 mg/kg), while also demonstrating superiority at specific time points (*P* < 0.05). Notably, water consumption slightly decreased post-treatment compared to the vehicle group. Furthermore, there were significant increases in natriuresis and diuresis observed on the first day of treatment with 23 mg/kg and 68 mg/kg S086 (*P* < 0.001). However, over the course of treatment, the effects in all treatment groups gradually diminished. This study demonstrates the anti-hypertensive efficacy of S086 in DSS hypertensive rat model, offering promising avenues for the clinical development of S086 as a hypertension treatment.

## Graphical Abstract

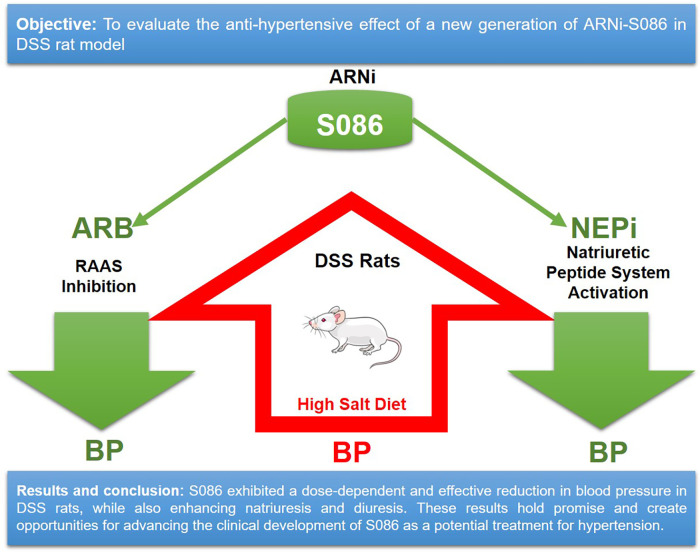

## 1 Introduction

Hypertension is a worldwide chronic cardiovascular disease (CVD) and the leading cause for premature death. Over the past few decades, the population of hypertension patients has surged due to the aging of society (1). According to data from the NCD (non-communicable diseases) Risk Factor Collaboration, there were more than 1.2 billion people aged 30–79 with hypertension worldwide in 2019, double the number from 1990 (2). Notably, the 2017 American College of Cardiology (ACC)/American Heart Association (AHA) Hypertension Guidelines have lowered the threshold for hypertension diagnosis from a systolic blood pressure (BP)/diastolic BP of ≥140/90 mm Hg to ≥130/80 mm Hg, indicating an increase in patients needing medical treatment in the future (3). High salt dietary consumption is one of the predominant risk factors for the essential pathogenesis of hypertension and strongly correlated with BP-independent organ damage. It has been reported that 30%–50% of hypertensive patients are associated with high salt intake (4). Studies suggest that high salt intake can lead to increased blood pressure by decreasing glomerular filtration membrane permeability, reducing filtration area and the number of glomeruli, and producing excess reactive oxygen species. As sodium load increases, endothelial function decreases, resulting in impaired vascular relaxation and elevated blood pressure (5, 6). Diuretics are effective in lowering blood pressure caused by a high-salt diet, but their adverse effects, such as low potassium and high uric acid, also require attention in clinics (7).

ARNi, which is a co-crystal comprising an angiotensin receptor II blocker (ARB) and a neprilysin inhibitor (NEPi), is a novel therapy for cardiovascular disease (8). Its most common use in clinical practice is for heart failure patients, particularly those with reduced ejection fraction (HF-REF), and is recommended as the first-line therapy for HF-REF treatment by ACC (American College of Cardiology)/AHA (American Heart Association)/HFSA (Heart Failure Society of America) and ESC (European Society of Cardiology) guidelines (9, 10). Additionally, ARNi has shown efficacy in hypertensive patients due to NEPi's mechanism of inhibiting ANP (Atrial Natriuretic Peptide) and BNP (Brain natriuretic peptide) degradation which leads to natriuresis and diuresis, potentially reducing blood pressure in salt-sensitive patients (11). LCZ696 (Entresto) is the first ARNi product launched globally and has shown profound efficacy in controlling BP in Asian patients with salt-sensitive hypertension (12). In several clinical trials, LCZ696 had a significantly superior effect on BP control compared with current first-line antihypertensive agents, Valsartan, and Olmesartan (13–16).

S086, a novel ARNi which is a co-crystal containing ARB (an active metabolite of Losartan), NEPi, calcium, and hydrate. EXP3174, which is a higher potency and longer t_1/2_ ARB in S086 than Valsartan in LCZ696. The NEPi ingredient in S086 is the same as that in LCZ696, which is a prodrug of LBQ657-Sacubitril. S086 has been proven to have comparable efficacy to LCZ696 in rat and dog myocardial infarction models. Both S086 and LCZ696 improved the left ventricular ejection fraction and myocardial fibrosis, and do not have a significant impact on hemodynamics (17). The phase I clinical trial for S086 in healthy volunteers indicated a dose-dependent pharmacokinetic profile (C_max_ and AUC) and pharmacodynamic effect (mean diastolic and systolic blood pressure). Throughout the duration of the trial, there were no reports of serious adverse events. Hypotension was the most commonly observed side effect, which is an expected pharmacological reaction to S086 (18).

To further advance the clinical applications of S086, we conducted a study utilizing an implantable telemetry system to observe real-time blood pressure. We investigated the antihypertensive effects of the new generation of ARNi in a rat model of Dahl Salt Sensitive (DSS) hypertension and measured natriuresis and diuresis. In this study, we found that S086 demonstrates a dose-dependent antihypertensive effect and greater potency in controlling blood pressure compared to LCZ696, EXP3174, and Sacubitril. These results are promising and open up opportunities for further clinical investigations of S086 as a potential hypertension treatment.

## 2 Materials and methods

### 2.1 Animals

Dahl Salt Sensitive (DSS) male rats aged 7–9 weeks were provided by Beijing Vital River Laboratory Animal Technology Co., Ltd. The animals were free to access sterilized standard laboratory food and water. The animal room environment was controlled (target conditions: temperature 20 to 26°C, relative humidity 30%–70%, 12 h of artificial light, and 12 h of dark). Temperature and relative humidity were monitored twice daily. The study was conducted in accordance with the Institutional Animal Care and Use Committee at WuXi AppTec.

### 2.2 Reagents

All the test drugs were supplied by Huizhou Salubris Co. Ltd (Storage condition: 25°C nitrogen cabinet free of light): S086 (Lot: ZXM18002, Purity: 95.51%), EXP3174 (Lot: SXMA18002, Purity: 91.47%), Sacubitril calcium salt (Lot: SXMA18001, Purity: 95.36%), and LCZ696 (Lot: DYF17006, Purity: 94.37%).

The study used 0.5% CMC-Na (Sodium Carboxymethyl Cellulose) as the vehicle for all the compounds. This vehicle was prepared by weighing and dissolving CMC-Na in deionized water at a concentration of 0.5 g CMC-Na/100 ml water. All the compounds were dissolved in this 0.5% CMC-Na solution at a concentration calculated based on their anhydrous-free acid weight. The theoretical weight of each compound was multiplied by a corresponding conversion factor (S086: 1.12; Sacubitril calcium salt: 1.10; EXP3174: 1.09; and LCZ696: 1.14) to determine the actual weight to be used in the solution. The prepared solutions were stored at 2–8°C and were used within specific timeframes. S086 and EXP3174 solutions were prepared every 3 days, while Sacubitril calcium salt and LCZ696 solutions were prepared daily. Prior to dosing, the solutions were allowed to reach room temperature for 10–15 min. The rats were each given a dosage of 5 ml/kg of body weight, which was well-mixed with their respective solutions.

Pentobarbital was purchased from Alfasan International B.V and stored at room temperature. Na^+^ test kit was purchased from Medical System and stored at 2–8°C. 8% salt forage (blue particles) and 0.3% salt forage (yellow particles) were purchased from Jiangsu Medicience and stored at 2–8°C. Meloxicam injection was purchased from Qilu Animal Health Products CO., LTD. and stored at room temperature. Gentamicin was purchased from Yichang Humanwell Pharma CO. and stored at room temperature.

### 2.3 Implantation of blood pressure telemetry implant

The rats were injected intraperitoneally with a pentobarbital-normal saline solution (50 mg/kg) for anesthesia before undergoing the implantation procedure, which involved the following steps: performing an abdominal incision surgery on the rats and separating their abdominal aorta; injecting a blood pressure detection probe into the abdominal aorta and securing it to the abdominal wall; suturing the muscles and skin and administering subcutaneous injections of meloxicam (1 mg/kg) for pain relief and gentamicin (5 mg/kg) for infection prevention; placing the rats on a constant temperature blanket and feeding them separately after they regained consciousness. Additionally, subcutaneous injections of meloxicam (1 mg/kg) for pain relief and gentamicin (5 mg/kg) for infection prevention were also administered daily for three days after surgery.

### 2.4 Dahl salt sensitive hypertensive rat model

**Figure 1:**
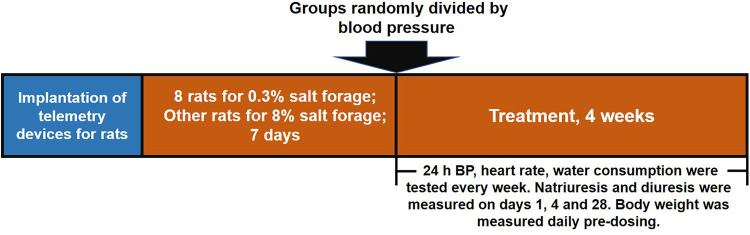
Schematic design of the study. The study was divided into three stages. Firstly, telemetry devices were implanted into rats. Secondly, DSS rat hypertension model was created by administering high salt diets for 7 days and sham group animals were administered low salt diets. Thirdly, rats were treated with the designated interventions for 4 weeks, and body weight, blood pressure, heart rate, water consumption, natriuresis, and diuresis were measured during the treatment period.

Telemetry devices were implanted in rats to measure heart rate and 24-h baseline blood pressure. The rats were divided into two groups, with one group receiving 0.3% salt forage as a sham and the other group receiving 8% salt forage to induce hypertension. After seven days, rats with an average 24-h systolic blood pressure of ≥160 mmHg were chosen and randomly divided into seven groups based on their systolic blood pressure. The successful standard for generating a hypertensive model was an average 24-h systolic blood pressure of ≥160 mmHg. The groups included sham group (*n* = 8), vehicle group (*n* = 7), LCZ696 group (68 mg/kg, *n* = 7), EXP3174 group (35 mg/kg, *n* = 7), Sacubitril group (33 mg/kg, *n* = 7), S086 low dose group (8 mg/kg, *n* = 7), S086 middle dose group (23 mg/kg, *n* = 7), and S086 high dose group (68 mg/kg, *n* = 7). The LCZ696, EXP3174, Sacubitril, and S086 high dose groups received equimolar doses of compounds. All groups were orally administered for 28 days, and dosing occurred between 11:00 AM and 12:00 PM daily. The rats’ heart rate and blood pressure were monitored weekly (Figure 1).

### 2.5 Blood pressure and heart rate detection

The Dataquest ART system by Data Sciences International was utilized for real-time monitoring of blood pressure and heart rate for 24 h on days 1, 7, 14, 21, and 28 after administering, with 11:00 AM data serving as a baseline each day (refer to Figure 1). The analysis of blood pressure was performed using Ponemah Software 5.0 from Data Sciences International, and the MAP (Mean Arterial Pressure) was calculated using the following formula: MAP = (SBP + 2 × DBP)/3.

### 2.6 Water consumption, natriuresis and diuresis detection

Water consumption and urinary output were measured using metabolic cages. Rats were placed in the metabolic cages at 16:00 PM∼17:00 PM on the day before detection day. Urine was collected 6 h after dosing for the assessment of natriuresis and diuresis on days 1, 4 and 28. 24-h water consumption was recorded and measured twice weekly. Natriuresis was quantified using Na^+^ test kits and a fully automatic biochemical instrument from HITACHI Automatic Analyzer 3100 (19).

### 2.7 Body weight

Body weight was measured daily before dosing.

### 2.8 Statistical analysis

The manuscript complies with British Journal of Pharmacology's recommendations and requirements on experimental design and analysis (20). The declared group size is the number of independent values, and statistical analysis was done using these independent values (i.e., not treating technical replicates as independent values). In terms of this study, data were presented as means ± S.E.M. Plots were produced using Graphpad Prism 9.0. Statistical analysis was performed using the SPSS software. For multiple groups comparison, the Levene's test was used to test for equality of variances; if *P* > 0.05, one-way ANOVA were performed; if *P* ≤ 0.05, Kruskal–Wallis test were performed. For one-way ANOVA, if *P* ≤ 0.05, the LSD *post hoc* test was performed. For the Kruskal–Wallis test, if *P* ≤ 0.05, the LSD *post hoc* test was performed after the ranks were transformed into normal scores. Otherwise, no data normalization was performed. We conducted a full data analysis without excluding outliers. A *P*-value <0.05 was considered statistically significant.

## 3 Results

### 3.1 S086 effectively reduced systolic blood pressure (SBP) in DSS rats

**Figure 2:**
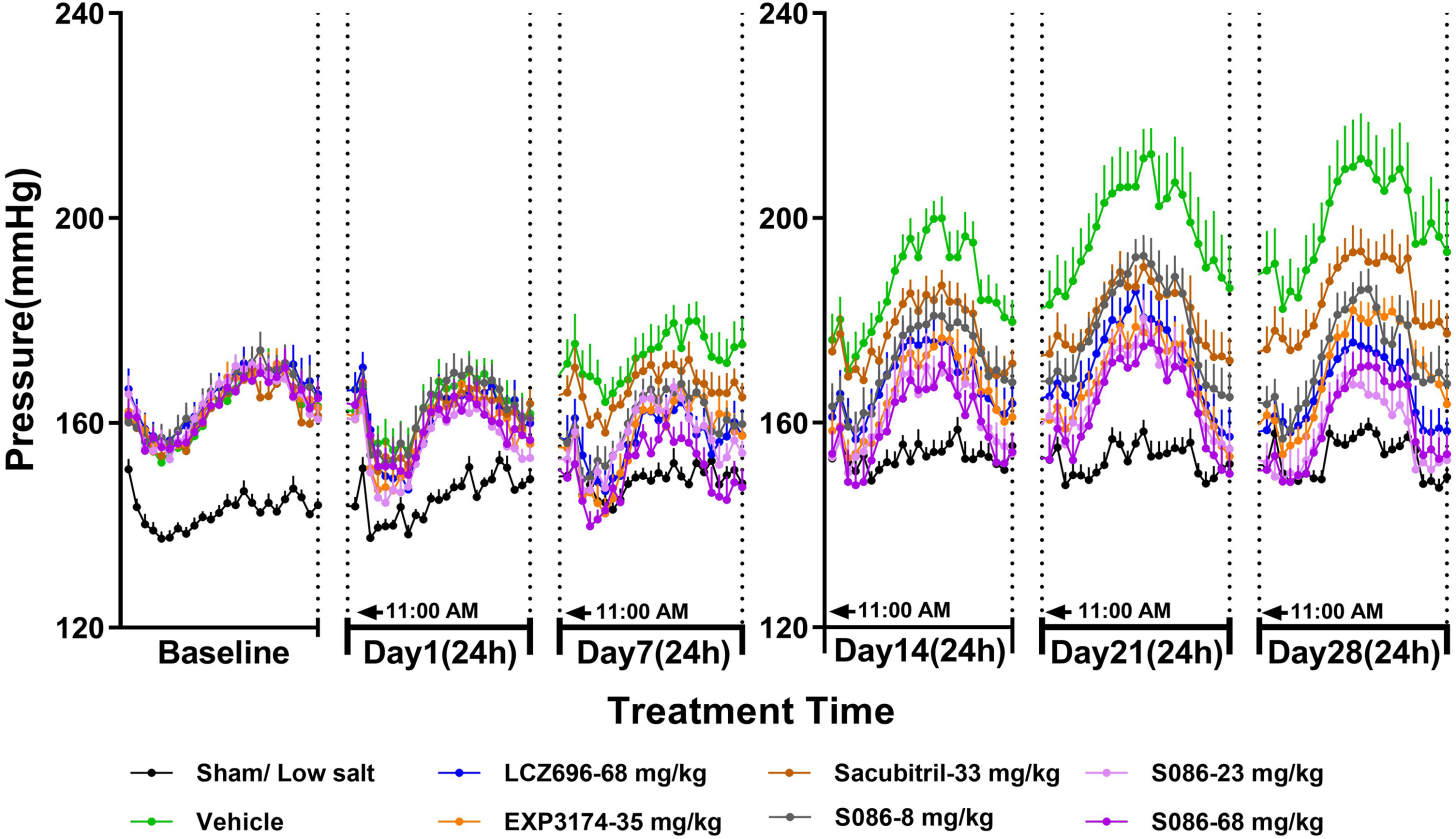
The 24-h systolic blood pressure (SBP) and time curve. The successfully established DSS rats were randomly divided into seven groups (*n* = 7/group), with each group receiving different drugs or the same drug with different doses, as indicated in the figure. The low salt group (*n* = 8/group) was used as sham control. The vehicle model group rats that received solvent. SBP was tested every week.

The SBP in DSS rats increased progressively over time in the vehicle high salt (8%) group, and was significantly higher compared to the sham low salt (0.3%) group at each measurement point between day 1 and day 28 after dosing (*P* < 0.001). The SBP in the sham low salt (0.3%) group was approximately 150 mmHg, whereas the SBP in the vehicle high salt (8%) group ranged from 162.87 mmHg on day 1 to 199.15 mmHg on day 28 (Table 1). Notably, the peak SBP occurred between 9:00 PM and 2:00 AM, while the valley SBP was observed between 1:00 PM and 6:00 PM (Figure 2).

**Table 1:** The 24-h mean SBP on Days 1, 7, 14, 21, 28 (*n* ≥ 7, mean ± sem).

Group	*n*	SBP (mmHg)					
		Baseline	Day 1	Day 7	Day 14	Day 21	Day 28
Sham/Low salt	8	142.67 ± 1.29	145.26 ± 1.25	148.61 ± 1.52	153.51 ± 2.07	152.66 ± 2.15	153.42 ± 1.52
Vehicle	7	162.33 ± 3.41^##^	162.87 ± 3.50^##^	172.82 ± 3.64^###^	186.61 ± 3.82^###^	198.07 ± 7.40^###^	199.15 ± 7.45^###^
LCZ696 68 mg/kg	7	165.03 ± 2.99	160.14 ± 2.98	156.64 ± 3.59***	168.23 ± 4.77**	172.15 ± 4.81***	166.35 ± 4.28***
EXP3174 35 mg/kg	7	163.52 ± 2.61	158.23 ± 3.01	157.07 ± 2.67	165.90 ± 3.31**	169.15 ± 2.86***	170.42 ± 2.69***
Sacubitril 33 mg/kg	7	161.81 ± 3.05	160.76 ± 2.52	166.75 ± 2.02	177.55 ± 2.66	181.12 ± 4.05**	184.29 ± 4.19*
S086 8 mg/kg	7	164.52 ± 2.88	162.65 ± 2.81	159.85 ± 2.11*	171.67 ± 3.94*	179.16 ± 4.24**	172.75 ± 3.64***
S086 23 mg/kg	7	163.76 ± 1.41	155.94 ± 1.99	157.06 ± 1.66**	161.81 ± 5.23***	166.70 ± 3.44***	157.61 ± 5.13***
S086 68 mg/kg	7	162.84 ± 2.48	158.59 ± 3.30	150.26 ± 2.81^***,^,&&^	159.93 ± 4.56^***,&^	164.20 ± 4.52^***,&&^	160.32 ± 2.90^***,^,&&&^

^##^ *P* < 0.01, ^###^*P* < 0.001 vs. Sham.

* *P* < 0.05, **P < 0.01, ***P < 0.001 vs. Vehicle.

^^^ *P* < 0.05, ^^^^*P* < 0.01 vs. EXP3174.

^&^ *P* < 0.05, ^&&^*P* < 0.01, ^&&&^*P* < 0.001 vs. Sacubitril.

On day 1, there were no significant changes in SBP observed in each dosing group compared to the vehicle high salt (8%) group. However, on day 7, the LCZ696-68 mg/kg group, EXP3174-35 mg/kg group, and different doses of S086 (8, 23, 68 mg/kg) groups showed significant reductions in the 24-h mean SBP compared to the vehicle high salt (8%) group. The reductions were 16.18 mmHg (*P* < 0.001), 15.75 mmHg (*P* < 0.01), 12.97 mmHg (*P* < 0.05), 15.76 mmHg (*P* < 0.01), and 22.56 mmHg (*P* < 0.001), with corresponding reduction rates of 9.4%, 9.1%, 7.5%, 9.1%, and 13.1%, respectively. S086 demonstrated dose-dependent efficacy in SBP and superior efficacy compared to an equal molar dose of LCZ696-68 mg/kg at specific time points (*P* < 0.05). Additionally, S086 had a significantly better effect on SBP compared to the equimolar dose of EXP3174 (*P* < 0.05) and Sacubitril (*P* < 0.001) (Table 1).

The efficacy of each dosing group on days 14, 21 and 28 showed similar results compared to day 7, and the efficacy increased over time compared to the vehicle high salt (8%) group. The reduction rate of SBP in LCZ696 group increased from 9.4% on day 7 to 16.5% on day 28, and from 13.1% to 19.5% for S086-68 mg/kg. The Sacubitril- 33 mg/kg group exhibited a significant effect on day 21 and 28, with the mean 24 h SBP reduced by 16.91 mmHg (*P* < 0.01) and 14.86 mmHg (*P* < 0.05) compared to the vehicle high salt (8%) group.

The middle and high dose groups of S086 (23 and 68 mg/kg) could sustain SBP levels similar to the sham group for approximately 14 h (11:00 AM to 8:00 PM and 6:00 AM to 11:00 AM) after 28 days of dosing. The other groups were unable to lower their SBP to the level of the sham group. (Figure 2)

### 3.2 S086 effectively reduced diastolic blood pressure (DBP) in DSS rats

**Figure 3:**
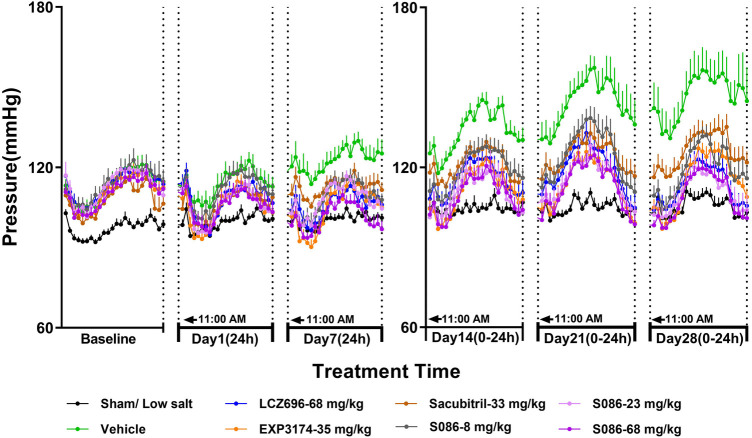
The 24-h diastolic blood pressure (DBP) and time curve. The successfully established DSS rats were randomly divided into seven groups (*n* = 7/group), with each group receiving different drugs or the same drug with different doses, as indicated in the figure. The low salt sham group (*n* = 8/group) was used as sham control. The vehicle model group rats that received solvent. DBP was tested every week.

The DBP in DSS rats followed a similar trend to the SBP results. In the sham low-salt (0.3%) group, the DBP was approximately 100 mmHg, while in the vehicle high-salt (8%) group, it increased from 113.87 mmHg on day 1 to 146.31 mmHg on day 28 (Figure 3, Table 2).

**Table 2:** The 24-h mean DBP on Days 1, 7, 14, 21, 28 (*n* ≥ 7, mean ± sem).

Group	*n*	DBP (mmHg)					
		Baseline	Day 1	Day 7	Day 14	Day 21	Day 28
Sham/Low salt	8	97.01 ± 1.46	99.52 ± 1.57	100.20 ± 1.40	104.46 ± 1.99	104.84 ± 1.83	105.68 ± 2.11
Vehicle	7	113.25 ± 2.80^##^	113.87 ± 3.23^##^	122.43 ± 3.52^###^	133.56 ± 3.07^###^	144.13 ± 6.48^###^	146.31 ± 8.36^###^
LCZ696 68 mg/kg	7	113.11 ± 2.99	107.74 ± 2.77	105.74 ± 2.91***	115.75 ± 4.44**	119.81 ± 4.63***	113.39 ± 3.80***
EXP3174 35 mg/kg	7	110.89 ± 2.30	105.07 ± 2.50	104.68 ± 2.28***	111.43 ± 3.29***	114.47 ± 2.92***	114.71 ± 2.63***
Sacubitril 33 mg/kg	7	108.08 ± 2.78	106.39 ± 2.88	112.69 ± 1.95*	121.27 ± 2.38*	124.47 ± 4.16**	126.10 ± 4.20**
S086 8 mg/kg	7	113.73 ± 3.63	111.08 ± 3.42	108.85 ± 2.85*	119.11 ± 3.91*	125.36 ± 4.83**	118.99 ± 3.67***
S086 23 mg/kg	7	112.77 ± 0.79	105.68 ± 1.92	107.83 ± 1.08*	110.77 ± 4.61***	115.36 ± 3.06***	110.20 ± 4.83***
S086 68 mg/kg	7	110.03 ± 2.50	105.45 ± 3.10	101.41 ± 2.13^***,&&^	109.38 ± 3.87^***,&&^	113.33 ± 3.86^***,&^	109.11 ± 2.45^***,&&^

^##^ *P* < 0.01, ^###^*P* < 0.001 vs. Sham.

* *P* < 0.05, **P < 0.01, ***P < 0.001 vs. Vehicle.

^&^ *P* < 0.05, ^&&^*P* < 0.01, ^&&&^*P* < 0.001 vs. Sacubitril.

On day 7, 24-h mean DBP decreased by 16.69 mmHg (*P* < 0.001), 17.75 mmHg (*P* < 0.001), 9.74 mmHg (*P* < 0.05), 13.58 mmHg (*P* < 0.05), 14.60 mmHg (*P* < 0.05), and 21.02 mmHg (*P* < 0.001) for the LCZ696-68 mg/kg group, EXP3174-35 mg/kg group, Sacubitril-33 mg/kg group, and different doses of S086 (8, 23, 68 mg/kg) groups. The reduction rates were 13.6%, 14.5%, 8.0%, 11.1%, 11.9%, and 17.2%, respectively (Table 2).

The middle and high dose groups of S086 (23 and 68 mg/kg) could sustain the DBP at or near the level of the sham group (some time points lower than the sham group) for about 14 h (from 11:00 AM to 8:00 PM and from 6:00 AM to 11:00 AM) after 28 days of dosing (Figure 3).

### 3.3 S086 effectively reduced mean arterial pressure (MAP) in DSS rats

**Figure 4:**
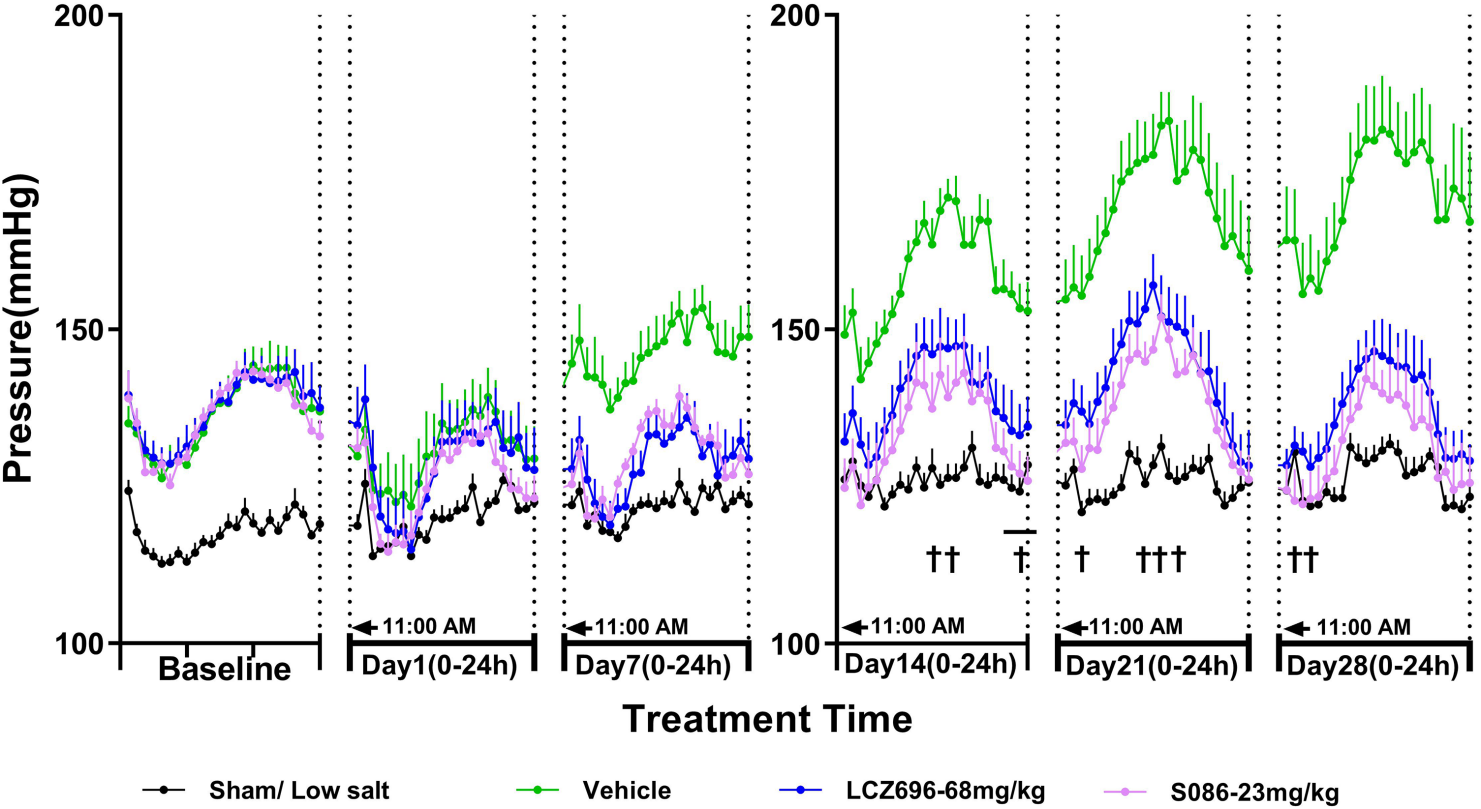
The 24-h mean arterial pressure (MAP) and time curve. MAP is calculated using the following formula: MAP = (SBP + 2×DBP)/3. ^†^*P* < 0.05 vs. LCZ696-68 mg/kg.

As MAP is calculated from SBP and DBP, the effect of each treatment group on MAP was similar to its effect on SBP and DBP. The MAP in the sham low-salt (0.3%) group was approximately 120 mmHg. The vehicle high-salt (8%) group had significantly higher MAP than the sham low-salt (0.3%) group at all time-points from day 1 to day 28 after dosing (*P* < 0.001). Efficacy of S086 middle dose (23 mg/kg) exhibited a non-inferiority compared to LCZ696-68 mg/kg and superior efficacy at specific time points (*P* < 0.05) (Figure 4, Table 3).

**Table 3:** The 24-hour mean MAP on Days 1, 7, 14, 21, 28 (*n* ≥ 7, mean ± sem).

Group	*n*	MAP (mmHg)					
		Baseline	Day 1	Day 7	Day 14	Day 21	Day 28
Sham/Low salt	8	117.44 ± 1.32	119.86 ± 1.38	121.56 ± 1.40	126.08 ± 1.99	125.84 ± 1.82	126.57 ± 1.82
Vehicle	7	136.24 ± 3.05^##^	130.53 ± 5.15^#^	146.10 ± 3.61^###^	158.37 ± 3.36^###^	169.50 ± 6.89^###^	170.77 ± 7.77^###^
LCZ696 68 mg/kg	7	136.91 ± 2.86	128.12 ± 5.46	128.54 ± 3.25***	139.41 ± 4.69**	143.39 ± 4.79***	137.00 ± 4.11***
S086 23 mg/kg	7	136.18 ± 0.89	125.52 ± 2.65	129.91 ± 1.17**	133.77 ± 4.87***	138.47 ± 3.32***	131.44 ± 4.99***

^#^ *P* < 0.05, ^##^*P* < 0.01, ^###^*P* < 0.001 vs. Sham.

* *P* < 0.05, **P < 0.01, ***P < 0.001 vs. Vehicle.

### 3.4 No significant change on heart rate (HR) in DSS rats

**Figure 5:**
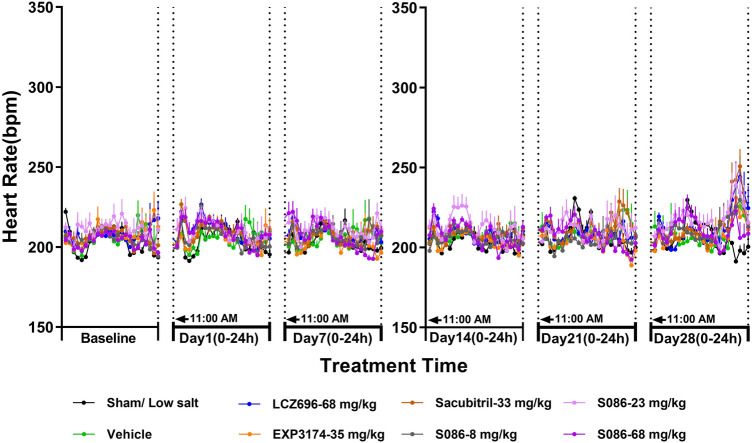
Heart rate (HR). The successfully established DSS rats were randomly divided into seven groups (*n* = 7/group), with each group receiving different drugs or the same drug with different doses, as indicated in the figure. The low salt sham group (*n* = 8/group) was used as sham control. The vehicle model group rats that received solvent. HR was tested every week.

There was no significant change in HR between each high-salt (8%) group (207.77 ± 3.17 bpm) and the sham group (206.26 ± 0.95 bpm) on day 28. The heart rates observed for the LCZ696 group, EXP3174 group, Sacubitril group, and the low-, middle-, and high-dose S086 groups were 211.98 ± 2.32, 208.36 ± 2.32, 213.17 ± 1.93, 211.32 ± 1.90, 213.30 ± 2.81, and 210.06 ± 2.72, respectively. Across all groups, no changes of clinical significance were noted (Figure 5).

### 3.5 Water consumption, natriuresis and diuresis

**Figure 6:**
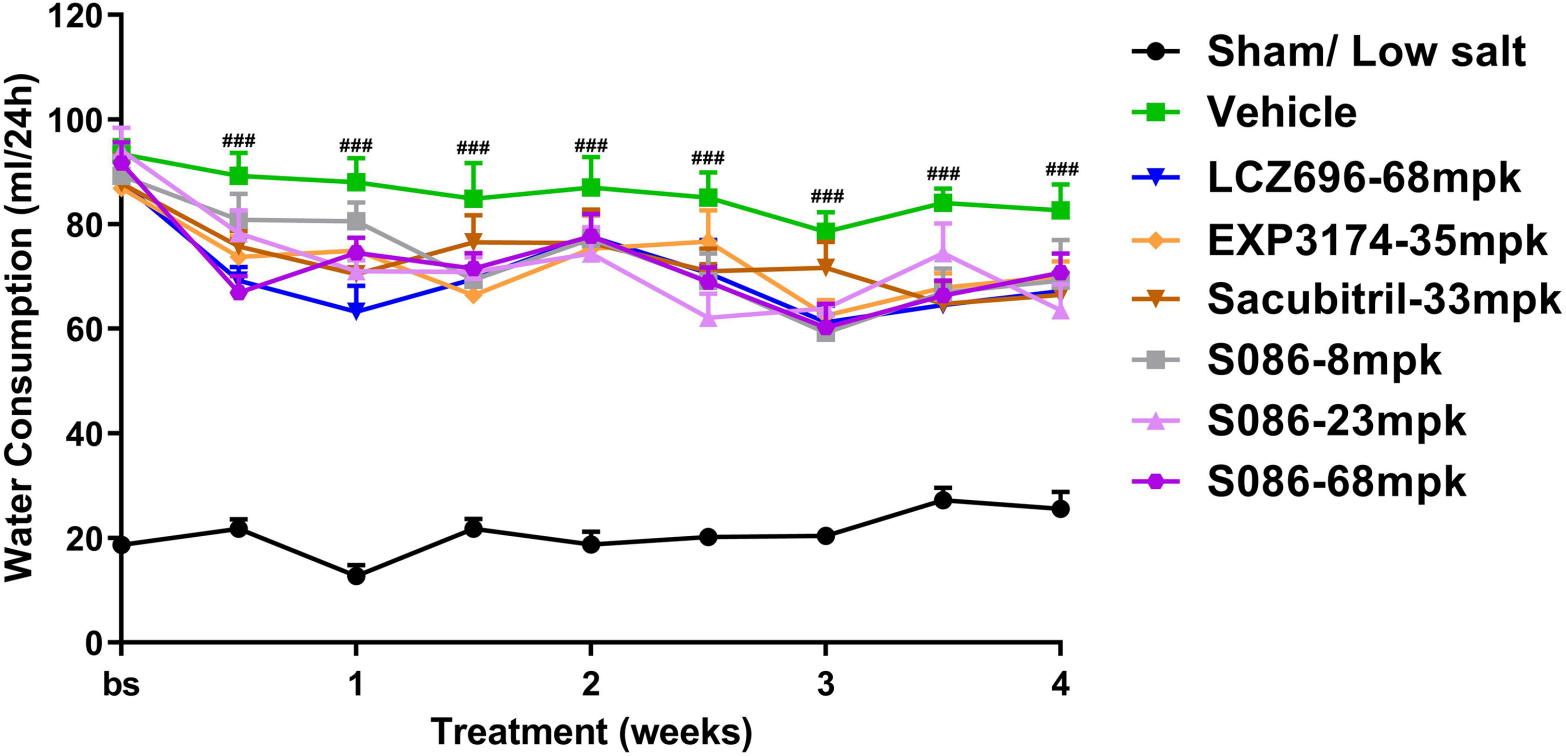
Water consumption. The successfully established DSS rats were randomly divided into seven groups (*n* = 7/group), with each group receiving different drugs or the same drug with different doses, as indicated in the figure. The low salt sham group (*n* = 8/group) was used as sham control. The vehicle model group rats that received solvent. Water consumption was tested every week. ^###^*P* < 0.001 vs. Sham.

**Figure 7:**
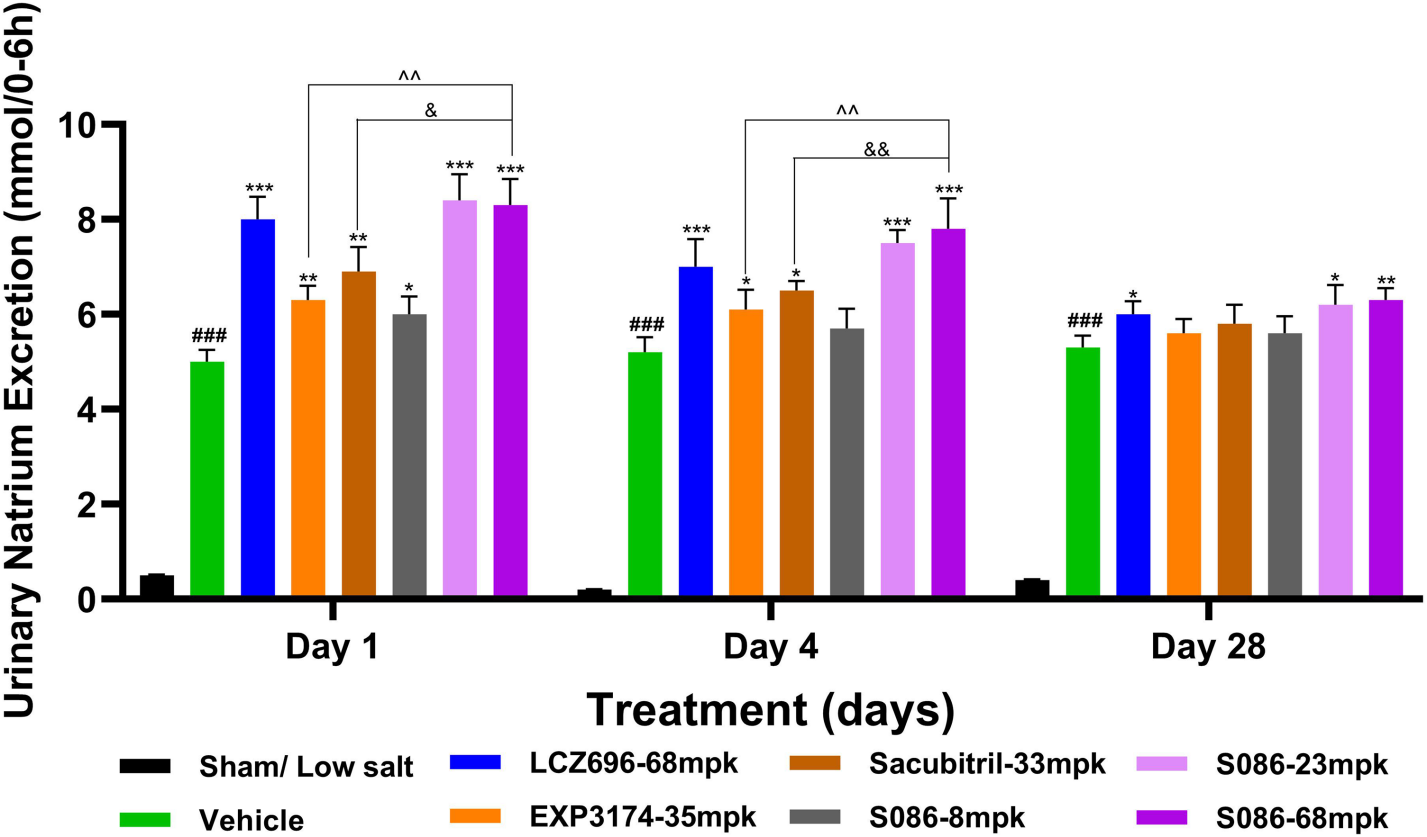
Natriuresis. The successfully established DSS rats were randomly divided into seven groups (*n* = 7/group), with each group receiving different drugs or the same drug with different doses, as indicated in the figure. The low salt sham group (*n* = 8/group) was used as sham control. The vehicle model group rats that received solvent. Natriuresis was tested on days 1, 4 and 28, 6 hours after dosing. ^###^*P* < 0.001 vs Sham; *P < 0.05, **P < 0.01, ***P < 0.001 vs Vehicle; ^^*P* < 0.01 vs EXP3174; ^&^*P* < 0.05, ^&&^*P* < 0.01 vs Sacubitril.

**Figure 8:**
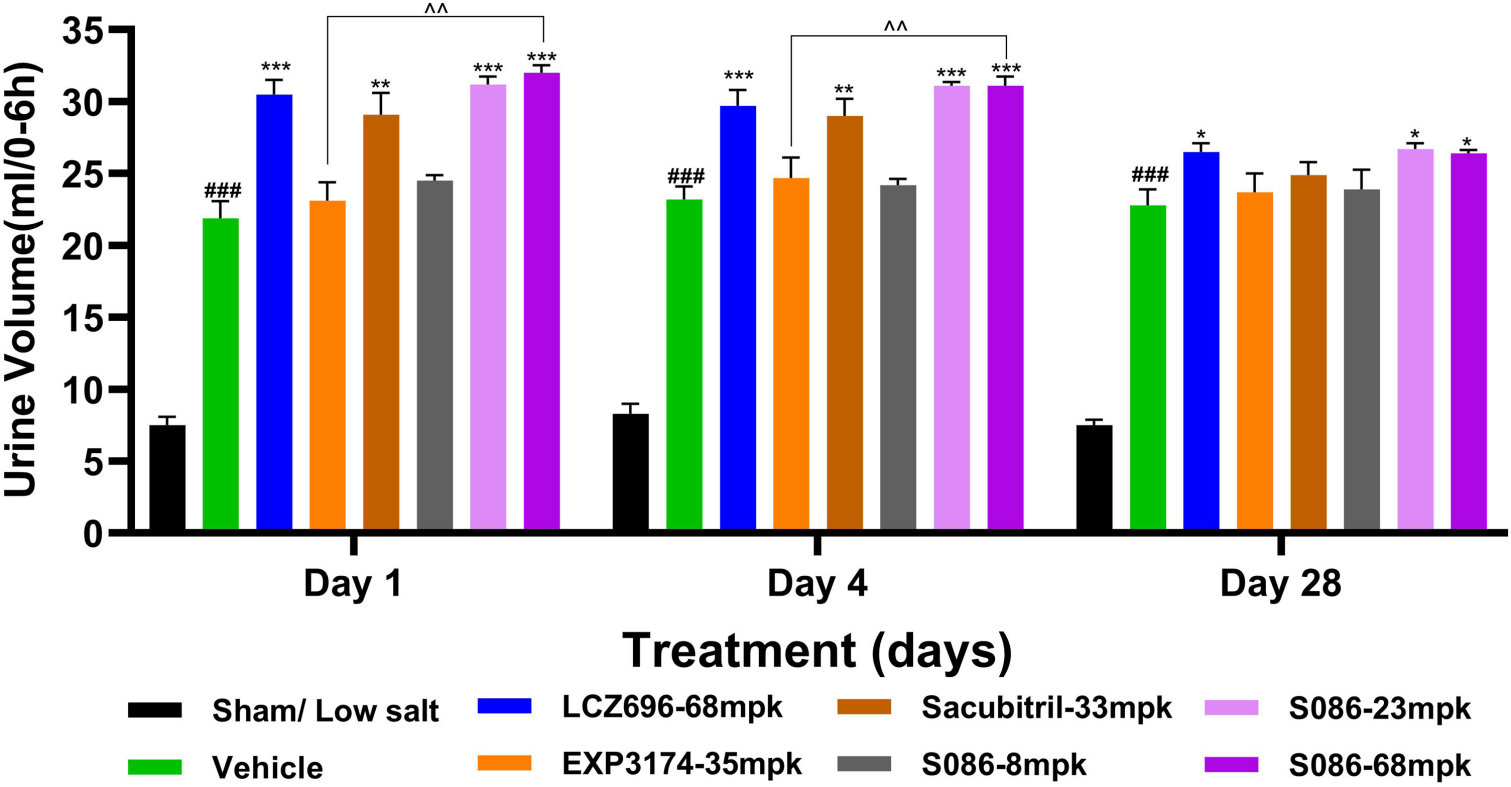
Diuresis. The successfully established DSS rats were randomly divided into seven groups (*n* = 7/group), with each group receiving different drugs or the same drug with different doses, as indicated in the figure. The low salt sham group (*n* = 8/group) was used as sham control. The vehicle model group rats that received solvent. Diuresis was tested on days 1, 4 and 28, 6 hours after dosing. ^###^*P* < 0.001 vs Sham; *P < 0.05, **P < 0.01, ***P < 0.001 vs Vehicle; ^^*P* < 0.01 vs EXP3174.

All high-salt (8%) groups exhibited significantly higher water consumption, urination, and urinary sodium excretion compared to the sham low-salt group (*P* < 0.001) (Figures 6–8).

Compared to the vehicle group, all treatment groups showed varying degrees of slight decrease in water consumption during the treatment time (Figure 6).

The results of the natriuresis study demonstrated that the strongest effect was observed on the first day of treatment, with significant differences compared to the vehicle group (*P* < 0.05). As the treatment duration progressed, the natriuretic effects of all treatment groups gradually diminished. However, on the 28th day of treatment, the LCZ696 group (*P* < 0.05) and the middle (*P* < 0.05)/high (*P* < 0.01)-dose groups of S086 still exhibited significant natriuretic effects (Figure 7).

Regarding diuresis, significant diuretic effects were observed on the first day of treatment in the LCZ696 group (*P* < 0.001), Sacubitril group (*P* < 0.01), and middle/high-dose groups of S086 (*P* < 0.001), with statistically significant differences. The EXP3174 group and the low-dose group of S086 showed an increasing trend in diuresis but without statistical significance. Over the course of treatment, the diuretic effects of all treatment groups gradually weakened. However, on the 28th day of treatment, the LCZ696 group and the middle/high-dose groups of S086 still exhibited significant diuretic effects (*P* < 0.05) (Figure 8).

### 3.6 Body weight

**Figure 9:**
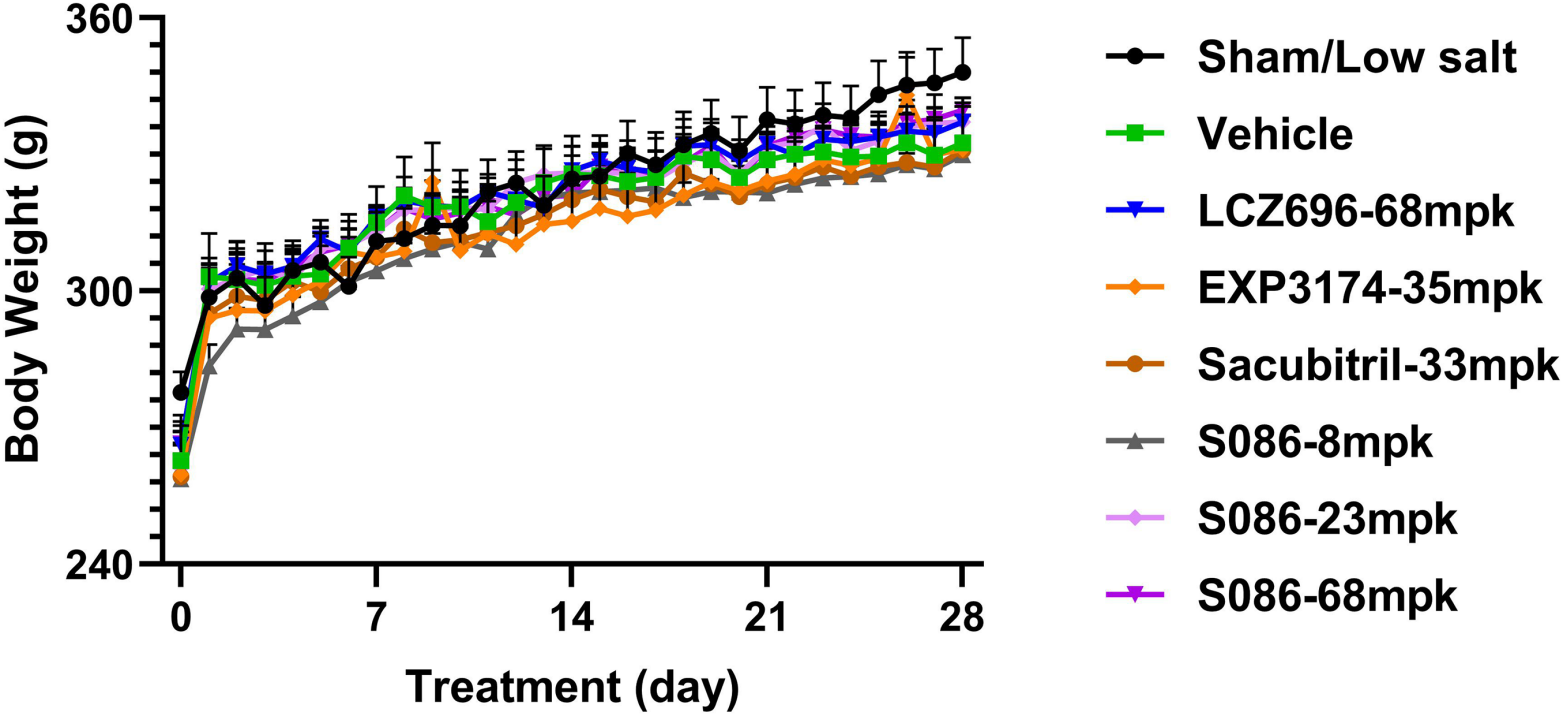
Body weight. The successfully established DSS rats were randomly divided into seven groups (*n* = 7/group), with each group receiving different drugs or the same drug with different doses, as indicated in the figure. The low salt sham group (*n* = 8/group) was used as sham control. Body weight was measured pre-dosing and daily after dosing.

Compared to the sham group, the vehicle group, EXP3174 group, and Sacubitril group all exhibited a significant decrease in body weight on day 28 (*P* < 0.05). However, no significant differences were observed between the vehicle group and all other treatment groups (Figure 9).

## 4 Discussion

Hypertension is a chronic cardiovascular disease characterized by elevated systolic and/or diastolic blood pressure. It is a major risk factor for various cardiovascular diseases. A high-salt diet is closely associated with hypertension, and its mechanism of action is complex. ARNi represents a novel class of antihypertensive medications, distinguished by its unique mechanism of action. This mechanism operates through the inhibition of both RAAS and NEP, which collectively contribute to blood pressure reduction. Specifically, the inhibition of RAAS results in vasodilation and a decrease in aldosterone secretion. Concurrently, NEP inhibition activates the natriuretic peptide system, leading to diuretic and natriuretic effects. The culmination of these effects ultimately results in the lowering of blood pressure (8). The first ARNi drug, LCZ696, has been reported to have significant antihypertensive effects, particularly in patients with salt-sensitive hypertension, and its clinical efficacy in reducing blood pressure is significantly better than that of Olmesartan, an angiotensin receptor blocker (ARB) (12). S086, a new generation of ARNi product developed by Salubris, has validated anti-heart failure effects in preclinical MI (Myocardial Infarction) chronic heart failure models and completed phase 1 clinical trial (17, 18). We investigated the antihypertensive effect of S086 compared to LCZ696 using the DSS rat model of hypertension and explored the diuretic and natriuretic effects of ARNi drugs. Additionally, we used a real-time telemetry system for blood pressure measurement, providing a more accurate reflection of rats' real-time blood pressure status, and avoiding blood pressure fluctuations caused by traditional animal manipulation methods.

Our study found that peak blood pressure values for both DSS rats and normal rats occurred between 9:00 PM and 2:00 AM, and trough values occurred between 1:00 PM and 6:00 PM. This indicates a significant difference from the circadian rhythm of human blood pressure, which peaks between 12:00 PM and 6:00 PM and has trough values between 1:00 AM and 4:00 AM according to clinical research (21, 22). Differences in blood pressure rhythms between rats and humans may be due to differences in their circadian activity rhythms. Rodents are typically active and eat at night and rest during the day, while humans are generally active and eat during the day and rest at night. Our administration of compounds to DSS rats at 11:00 AM is equivalent to humans taking medication before bedtime at 11:00 PM, which has certain guiding significance for the timing of administration in future rodent models of hypertension. If we aim to fully simulate human clinical medication habits (taking medication in the morning), we suggest administering animals from 5:00 PM to 6:00 PM (23, 24).

The new generation of ARNi drug-S086 demonstrated significant antihypertensive effect in the DSS rat model of hypertension, dose-dependently reducing both systolic and diastolic blood pressure (Figures 2, 3). In the DSS hypertensive rat model, a high-salt diet leads to sodium and water retention, resulting in increased blood volume and elevated blood pressure. Furthermore, studies have reported that a high-salt diet directly activates the renin-angiotensin-aldosterone system (RAAS), which is an additional mechanism contributing to the development of high blood pressure (25). S086, composed of an ARB and NEP inhibitor, can directly inhibit the RAAS system while activating the natriuretic peptide system, ultimately lowering blood pressure. Compared to LCZ696 (68 mg/kg), the middle dose of S086 (23 mg/kg) demonstrated efficacy comparable to LCZ696 (68 mg/kg), while also demonstrating superiority at specific time points (*P* < 0.05) (Figure 4). Prior studies had reported that the ARB component of S086-EXP3174 exhibited greater potency towards AT1 receptor and a longer half-life compared to Valsartan in LCZ696, which could potentially lead to clinical benefits for patients with hypertension (18, 26, 27). Additionally, the antihypertensive effects of S086 are better than those of an equimolar dose of EXP3174 for two reasons. Firstly, S086 metabolizes into EXP3174 and Sacubitril. Sacubitril further metabolizes into LBQ657- NEP inhibitor, and both of EXP3174 and LBQ657 reduce blood pressure through different mechanisms. Although the antihypertensive effect of the NEP inhibitor is relatively moderate, it is still stronger than that of the separate dose of EXP3174. Secondly, Sacubitril increases the exposure level of EXP3174, resulting in a higher exposure after administering an equimolar dose of S086 (17).

LBQ657 as a neprilysin inhibitor has been reported to significantly increase the expression of ANP and other natriuretic peptides in the body (28). Upon metabolism into LBQ657, S086 activates the natriuretic peptide system, resulting in natriuresis and diuresis (29). EXP3174, a high potent ARB metabolized from S086, has been shown to have a natriuretic effect (30). We investigated the effects of each compound on water consumption, natriuresis and diuresis in the DSS model. The results indicated that each treatment group showed a slight decrease in water consumption compared to the vehicle group. This decrease may be attributed to the drug's natriuretic and diuretic effects, leading to differences in salt and water balance in the body. Regarding the natriuresis and diuresis study, significant natriuretic and diuretic effects were observed in all treatment groups on the first dosing day (*P* < 0.05). However, over time, the intensity of these effects gradually diminished, which aligns with the trend observed in clinical studies of LCZ696 in patients with salt-sensitive hypertension. In that study, compared to Valsartan monotherapy, LCZ696 showed significant increases in natriuresis and diuresis on the first day after administration, which could not be sustained (12). However, we observed antihypertensive efficacy with Sacubitril (pro-drug of LBQ657) monotherapy, especially after 14∼28 days of treatment, when its effect was stronger than after 7 days. This suggests that the antihypertensive effect of NEPi may not be solely due to natriuresis and diuresis, but rather from vasodilation. ANP and BNP can activate receptors expressed in peripheral blood vessels, leading to vasodilation. ANP and BNP can also inhibit aldosterone release, blocking the downstream signaling pathway of the RAAS system, which finally lead to antihypertensive effect (31). However, the inhibitory effect of NEPi on the downstream signaling of the RAAS system activates the body's negative feedback regulation mechanism, promoting the activity of upstream signals of the RAAS system, thereby activating the RAAS system. Therefore, NEPi's antihypertensive effect alone is slight and must be used in combination with RAAS system blockers. The first-generation NEPi-omapatrilat simultaneously inhibited both NEP and ACE to activate the natriuretic peptide system and inhibit the RAAS system. However, due to the accumulation of bradykinin (a substrate of NEP and ACE) in the body, causing severe vascular edema, the drug was ultimately withdrawn from the market (32, 33).

NEPi and ARB combine to form a cocrystal, which reduces the risk of vascular edema caused by omapatrilat (NEPi and ACE). The cocrystal form has better drug properties than physical mixtures since it improves solubility, enhances compound pharmacokinetic properties, and increases absorption (34, 35). We developed S086-a novel ARNi cocrystal, which improved EXP3174's poor PK profile. Preclinical studies validated its significant blood pressure-reduction effect, superior to LCZ696. A completed phase 1 clinical trial demonstrated that S086 is well-absorbed in the human body, exhibits linear absorption, and can significantly affect target-related biomarkers. These provide a solid foundation for conducting further clinical studies. We will explore S086's antihypertensive effect in future phase 2 and phase 3 clinical trials, providing better treatment options for hypertension patients.

## 5 Conclusions

In our preclinical study on DSS rats with hypertension, we observed that the novel ARNi drug S086 had a significant antihypertensive effect. Its effect was potential to surpass that of the first-generation ARNi drug LCZ696. These results support further phase 2 and phase 3 clinical studies on S086 to explore its efficacy and safety in treating patients with hypertension.

## Data Availability

The raw data supporting the conclusions of this article will be made available by the authors, without undue reservation.

## References

[1] Mills KT Stefanescu A He J. The global epidemiology of hypertension. Nat Rev Nephrol. (2020) 16:223–37. 10.1038/s41581-019-0244-2 32024986 PMC7998524

[2] NCD Risk Factor Collaboration (NCD-RisC). Worldwide trends in hypertension prevalence and progress in treatment and control from 1990 to 2019: a pooled analysis of 1201 population-representative studies with 104 million participants. Lancet. (2021) 398:957–80. 10.1016/S0140-6736(21)01330-1 34450083 PMC8446938

[3] Martínez-Rueda AJ Olivas-Martínez A Vega-Vega O Fonseca-Correa JI Correa-Rotter R. New 2017 American College of Cardiology / American Heart Association high blood pressure guideline. Hypertension. (2019) 73:142–47. 10.1161/HYPERTENSIONAHA.118.11827 30571542

[4] Chatelanat O Pechère-Bertschi A Ponte B. Sensibilité au sel et hypertension artérielle [Salt sensitivity and hypertension]. Rev Med Suisse. (2019) 15:1625–28. . 31508914

[5] Di Palo KE Barone NJ. Hypertension and heart failure: prevention, targets, and treatment. Heart Fail Clin. (2020) 16:99–106. 10.1016/j.hfc.2019.09.001 31735319

[6] Cuka E Simonini M Lanzani C Zagato L Citterio L Messaggio E Inverse salt sensitivity: an independent risk factor for cardiovascular damage in essential hypertension. J Hypertens. (2022) 40:1504–12. 10.1097/HJH.0000000000003174 35881450

[7] Garfinkle MA. Salt and essential hypertension: pathophysiology and implications for treatment. J Am Soc Hypertens. (2017) 11:385–91. 10.1016/j.jash.2017.04.006 28479261

[8] Kuchulakanti PK. ARNI in cardiovascular disease: current evidence and future perspectives. Future Cardiol. (2020) 16:505–15. 10.2217/fca-2019-0089 32319309

[9] Yancy CW Jessup M Bozkurt B Butler J Casey DE Jr Colvin MM 2017 ACC/AHA/HFSA focused update of the 2013 ACCF/AHA guideline for the management of heart failure: a report of the American College of Cardiology/American Heart Association task force on clinical practice guidelines and the heart failure society of America. Circulation. (2017) 136:e137–61. 10.1161/CIR.0000000000000509 28455343

[10] Ponikowski P Voors AA Anker SD Bueno H Cleland JGF Coats AJS 2016 ESC guidelines for the diagnosis and treatment of acute and chronic heart failure: the task force for the diagnosis and treatment of acute and chronic heart failure of the European Society of Cardiology (ESC). developed with the special contribution of the Heart Failure Association (HFA) of the ESC. Eur J Heart Fail. (2016) 18:891–75. 10.1093/eurheartj/ehw128 27207191

[11] Yamamoto K Rakugi H. Angiotensin receptor-neprilysin inhibitors: comprehensive review and implications in hypertension treatment. Hypertens Res. (2021) 44:1239–50. 10.1038/s41440-021-00706-1 34290389

[12] Wang TD Tan RS Lee HY Ihm SH Rhee MY Tomlinson B Effects of sacubitril/valsartan (LCZ696) on natriuresis, diuresis, blood pressures, and NT-proBNP in salt-sensitive hypertension. Hypertension. (2017) 69:32–41. 10.1161/HYPERTENSIONAHA.116.08484 27849566

[13] Williams B Cockcroft JR Kario K Zappe DH Brunel PC Wang Q Effects of sacubitril/valsartan versus olmesartan on central hemodynamics in the elderly with systolic hypertension: the PARAMETER study. Hypertension. (2017) 69:411–20. 10.1161/HYPERTENSIONAHA.116.08556 28093466

[14] Supasyndh O Wang J Hafeez K Zhang Y Zhang J Rakugi H. Efficacy and safety of sacubitril/valsartan (LCZ696) compared with olmesartan in elderly Asian patients (≥65 years) with systolic hypertension. Am J Hypertens. (2017) 30:1163–69. 10.1093/ajh/hpx111 28992296

[15] Ruilope LM Dukat A Böhm M Lacourcière Y Gong J Lefkowitz MP. Blood-pressure reduction with LCZ696, a novel dual-acting inhibitor of the angiotensin II receptor and neprilysin: a randomised, double-blind, placebo-controlled, active comparator study. Lancet. (2010) 375:1255–66. 10.1016/S0140-6736(09)61966-8 20236700

[16] Ye L Wang J Chen Q Yang X. LCZ696, a promising novel agent in treating hypertension (a meta-analysis of randomized controlled trials). Oncotarget. (2017) 8:107991–05. 10.18632/oncotarget.22442 29296218 PMC5746120

[17] Sun J Xu W Hua H Xiao Y Chen X Gao Z Pharmacodynamic and pharmacokinetic effects of S086, a novel angiotensin receptor neprilysin inhibitor. Biomed Pharmacother. (2020) 129:110410. 10.1016/j.biopha.2020.110410 32570118

[18] Hu Y Zhang H Li X Mai J Yang L Yan J A randomized, double-blind, placebo-controlled, single, and multiple dose-escalation phase I clinical trial to investigate the safety, pharmacokinetic, and pharmacodynamic profiles of oral S086, a novel angiotensin receptor-neprilysin inhibitor, in healthy Chinese volunteers. Expert Opin Investig Drugs. (2021) 8:1–9. 10.1080/13543784.2021.1985464 34633260

[19] Romero M Caniffi C Bouchet G Costa MA Elesgaray R Arranz C Chronic treatment with atrial natriuretic peptide in spontaneously hypertensive rats: beneficial renal effects and sex differences. PLoS One. (2015) 10:e0120362. 10.1371/journal.pone.0120362 25774801 PMC4361555

[20] Curtis MJ Alexander S Cirino G Docherty JR George CH Giembycz MA Experimental design and analysis and their reporting II: updated and simplified guidance for authors and peer reviewers. Br J Pharmacol. (2018) 175:987–93. 10.1111/bph.14153 29520785 PMC5843711

[21] Morris CJ Hastings JA Boyd K Krainski F Perhonen MA Scheer FA Day/night variability in blood pressure: influence of posture and physical activity. Am J Hypertens. (2013) 26:822–30. 10.1093/ajh/hpt026 23535155 PMC3693479

[22] Parati G Staessen JA. Day-night blood pressure variations: mechanisms, reproducibility and clinical relevance. J Hypertens. (2007) 25:2377–80. 10.1097/HJH.0b013e3282f2d116 17984656

[23] Acosta J Bussi IL Esquivel M Höcht C Golombek DA Agostino PV. Circadian modulation of motivation in mice. Behav Brain Res. (2020) 382:112471. 10.1016/j.bbr.2020.112471 31958519

[24] Tahara Y Aoyama S Shibata S. The mammalian circadian clock and its entrainment by stress and exercise. J Physiol Sci. (2017) 67:1–10. 10.1007/s12576-016-0450-7 27084533 PMC5138246

[25] Carmichael CY Wainford RD. Hypothalamic signaling mechanisms in hypertension. Curr Hypertens Rep. (2015) 17:39. 10.1007/s11906-015-0550-4 25860531 PMC4392165

[26] Van Liefde I Vauquelin G. Sartan-AT1 receptor interactions: in vitro evidence for insurmountable antagonism and inverse agonism. Mol Cell Endocrinol. (2009) 302:237–43. 10.1016/j.mce.2008.06.006 18620019

[27] Han Y Ayalasomayajula S Pan W Yang F Yuan Y Langenickel T Pharmacokinetics, safety and tolerability of sacubitril/valsartan (LCZ696) after single-dose administration in healthy Chinese subjects. Eur J Drug Metab Pharmacokinet. (2017) 42:109–16. 10.1007/s13318-016-0328-3 26961539

[28] Singh JSS Burrell LM Cherif M Squire IB Clark AL Lang CC. Sacubitril/valsartan: beyond natriuretic peptides. Heart. (2017) 103:1569–77. 10.1136/heartjnl-2017-311295 28689178

[29] Campbell HT Lightfoot BO Sklar AH. Four-hour atrial natriuretic peptide infusion in conscious rats: effects on urinary volume, sodium, and cyclic GMP. Proc Soc Exp Biol Med. (1988) 189:317–24. 10.3181/00379727-189-42813 2849771

[30] Wong PC Hart SD Duncia JV Timmermans PB. Nonpeptide angiotensin II receptor antagonists. Studies with DuP 753 and EXP3174 in dogs. Eur J Pharmacol. (1991) 202:323–30. 10.1016/0014-2999(91)90274-t 1748155

[31] Salazar J Rojas-Quintero J Cano C Pérez JL Ramírez P Carrasquero R Neprilysin: a potential therapeutic target of arterial hypertension? Curr Cardiol Rev. (2020) 16:25–35. 10.2174/1573403X15666190625160352 31241018 PMC7062041

[32] Omapatrilat TR. Bristol-Myers Squibb. Curr Opin Investig Drugs. (2001) 2:1414–22. 11890357

[33] Dzielska-Olczak M. Omapatrilat–nowy lek dla chorych na nadciśnienie tetnicze i niewydolność serca [Omapatrilat–new drug for patients with hypertension and heart failure]. Pol Merkur Lekarski. (2005) 19:556–63. 16379325

[34] Kim H Jang S Kim IW. Enhanced dissolution of naproxen by combining cocrystallization and eutectic formation. Pharmaceutics. (2021) 13:618. 10.3390/pharmaceutics13050618 33923065 PMC8145234

[35] Cysewski P. In silico screening of dicarboxylic acids for cocrystallization with phenylpiperazine derivatives based on both cocrystallization propensity and solubility advantage. J Mol Model. (2017) 23:136. 10.1007/s00894-017-3287-y 28349342 PMC5368210

